# Comment on: “The impact of psychiatric utilisation prior to cancer diagnosis on survival of solid organ malignancies”

**DOI:** 10.1038/s41416-019-0493-7

**Published:** 2019-06-10

**Authors:** Alain Braillon

**Affiliations:** Department of Medicine, University Hospital, 80000 Amiens, France

**Keywords:** Ethics, Public health

Klaassen et al. showed that patients with psychiatric utilisation prior to cancer diagnosis showed increased mortality from cancer and all causes.^1^ However, stating these most vulnerable people are “more likely to engage in behaviours, such as alcoholism and smoking”^1^ deserves robust comment.

First, vulnerable people are more prone to be victims than aggressors. Among aggressors are agents of the industrial epidemics: tobacco, alcohol and processed foods; the former ranking first but the last ones being on the rise, as public policies protect economic interests rather than lay people interests. Patients with psychiatric utilisation are not actively involved in an unhealthy activity as suggested by the term “engage”.^1^ Moreover, it is a double punishment as the system does not care for its victims. Smoking cessation services are not operating appropriately for those with severe mental illness, despite the American Psychiatric Association having issued its first practice guideline for the treatment of patients with nicotine dependence in 1996. Mental health settings have traditionally been exempt from smoke-free policy.^2–4^ Enduring calls for raising the bar for adequate treatment shows how deep the inertia is.^5^ Nevertheless, treatments are effective and safe when proactive: (a) nicotine replacement therapy plus motivational interviewing and cognitive behavioural therapy;^6^ (b) varenicline.^7^ The failure of the system to provide appropriate care cannot be overlooked: patients with serious mental illness make quit attempts and have plans to quit as smokers without mental illness.^8^

Second, the little progress for implementing smoking cessation for patients with serious mental illness is not restricted to psychiatry.

(a) Only one-half of patients with cancer who smoke are counselled to quit, despite, on one hand that smoking cessation is the most important factor in the outcome (cancer treatment effectiveness, overall survival, risk of the second primary malignancy and quality of life), and on the other hand, counselling is almost not effective as proactive treatment is needed (see above).^9^ Indeed, counselling or promoting brief intervention, as recommended by guidelines, is a pathological misconception. Who would simply rely on counselling or a brief intervention for a patient with type 2 diabetes to obtain healthy living? Low-intensity interventions are a smokescreen for denying care. Motivational interviewing and then after pharmacological treatment with psychological support are most effective when (a) providing reassurance because many smokers wrongly believe that smoking with patches is more dangerous than without, overlooking the devastating effects of compensatory uptake when trying to reduce smoking without patches; (b) using the “belt and braces” strategy, combining nicotine patches (several 21/24-h patches being frequently needed) with oral “rescue” formulations of nicotine (i.e., sprays and lozenges) to suppress occasional cravings. Indeed, “The Five-Day Plan to Stop Smoking” is another devastating pathological misconception, leading to a programmed failure. I never ask a smoker to quit but only to take the treatment and increase the doses as needed; craving is just pain and suffering. When they will be relieved, they will quit without effort. The number of visits is not the issue; indeed, stopping tobacco before the age of 40 avoids more than 90% of the excess mortality caused by continuing smoking. It is worth it, as one smoker out of two will die from smoking.

(b) Oncology is not an exception. For example, for patients with coronary disease, half of those smoking at the time of an acute event will remain persistent smokers, this prevalence having not changed over two decades.^9^

(c) Worse, in 2012, the US Joint Commission proposed to hospitals to document tobacco-use status of patients a month after discharge, but gave up because almost no hospital did it.^9^ Similarly, how could the Cancer Registry in Ontario fail to collect data about smoking status?^1^ Even more as it collects data about “rurality”.

Adults with schizophrenia are 3.5 times more likely to die than the general population, mainly due to lung cancer, chronic obstructive pulmonary and cardiovascular diseases; standardised mortality ratios being 52:100,000 person-years for suicide versus 75 for lung cancer.^10^ Similarly, news reports of violent incidents involving people with schizophrenia increase the public belief in their dangerousness, while they are less frequently violent than the whole population. Patients with mental health problems are not “engagers in” unhealthy lifestyles. Misconceptions and social stigmas towards patients with mental health problems prevent social attendance and help-seeking behaviour.

## Data Availability

Not applicable.
